# Complete chloroplast genome of *Baccaurea ramiflora* and its phylogenetic analysis

**DOI:** 10.1080/23802359.2021.2010616

**Published:** 2022-01-18

**Authors:** Ying-feng Niu, Jin Liu

**Affiliations:** Yunnan Institute of Tropical Crops, Xishuangbanna, China

**Keywords:** *Baccaurea ramiflora*, Chloroplast genome, phylogenetic analysis

## Abstract

*Baccaurea ramiflora* Lour. is a popular tropical fruit tree, mainly grown in Myanmar, India, and other tropical or sub-tropical regions where it is commonly referred to as Myanmar grapes, Burmese grapes, or Latkan, respectively. Besides food, *B. ramiflora* is a traditional medicinal plant with several pharmaceutical effects. It is also a crucial component of Chinese Dai medicine. Here, the chloroplast genome of *B. ramiflora* was sequenced, assembled, and annotated. The complete chloroplast genome is 161,093 bp in length with a GC content of 36.71%. Additionally, it comprises a large single-copy region (LSC) of 89,503 bp, a small single-copy region (SSC) of 18,818 bp, and two inverted repeat regions (IRa and IRb) of 26,386 bp. In total, 128 genes were annotated, including 82 protein-coding genes, 37 tRNA genes, 8 rRNA genes, and 1 pseudogene. Phylogenetic analysis revealed that *B. ramiflora* is closely related to *Phyllanthus emblica*, *Glochidion chodoense,* and *Phyllanthus amarus*. This study provides useful genomic information for future phylogenetic studies of *B. ramiflora* and Phyllanthaceae family.

*Baccaurea ramiflora* Lour. 1866 is a distinctive tropical fruit tree belonging to the Phyllanthaceae family (or Euphorbiaceae, subfamily Phyllanthoideae). It is commonly referred to as Myanmar grapes (Talambedu et al. 2014), Burmese grapes, or Latkan. *B. ramiflora* is mainly grown in Myanmar, India, Thailand, Vietnam, Laos, Cambodia, Malaysia, and China. Besides, it is one of the most common fruit tree species in the Southern Yunnan tropical rainforests, particularly in the tropical seasonal rainforest of Xishuangbanna (Wen and Cai 2014). Its fruits are born in the tree trunk or old branch and exhibit many colors, such as red, yellow, purple, and white, with the unique ‘old stem flower’ phenomenon of the tropical rain forest trees. *B. ramiflora* is exploited for various uses (Goyal et al. 2013). The mature fruit is rich in sugar, vitamin, and many trace elements and can be directly consumed as fresh fruit or used as a raw material for jam production (Goyal et al. 2020).

Moreover, *B. ramiflora* is a traditional medicinal plant (Puja et al. 2020). Its leaves, fruits, stems, bark, and seeds are essential ingredients of many herbal prescriptions used to treat jaundice, constipation, indigestion, and cellulitis. Their extracts are also utilized as an antidote for snake venom, antiphlogistic, and anodyne against rheumatoid arthritis (Goyal et al. 2020). Additionally, *B. ramiflora* extracts exhibit hypolipidemic, hypoglycemic, antiviral, antioxidant, diuretic, and cytotoxic activities (Nesa et al. 2018). Notably, *B. ramiflora* is also an essential component of Chinese Dai medicine, which has been used for centuries (Lin et al. 2003). Given its rich nutrient content and diverse medicinal applications, *B. ramiflora* has received immerse focus from scientists recently. Until now, most studies on *B. ramiflora* relate to its medicinal functions and active ingredients, with limited reports on its genome. Chloroplast genome harbors abundant genetic information and is a vital part of the plant genome. Here, the chloroplast genome of *B. ramiflora* was sequenced, assembled, and annotated.

The young leaves of *B. ramiflora* were collected from the Xishuangbanna Tropical Flowers and Plants Garden (100.786521 E, 22.014646 N). High-quality genomic DNA was extracted from the leaves using the DNeasy Plant Mini Kit (Qiagen, Germany), as per the manufacturer’s instructions. The specimen and DNA were deposited in the herbarium and cryogenic sample library of the Yunnan Institute of Tropical Crops (http://www.yitc.com.cn, Dr. Jin Liu, liujin06@126.com) with voucher numbers YITC-2020-FZ-P-032 and D2020-FZ-P-032, respectively. The sequencing library was constructed based on Illumina with the inserted size of 350 bp. Paired-end (PE) sequencing was performed on the Illumina HiSeq 2500 platform (Illumina, San Diego, CA, USA), and 7.5 Gb raw data was obtained. The clean reads were assembled with SPAdes-3.5.0 (http://soap.genomics.org.cn/soapdenovo.html) based on sequence overlap and paired-end relationships. Sanger sequencing was used to verify the four boundaries of the IR region. Chloroplast genome annotation was performed by CpGAVAS2 (Shi et al. 2019) and ORF Finder (https://www.ncbi.nlm.nih.gov/orffinder/). Chloroplast genome assembly and annotation results were submitted to GenBank (http://www.ncbi.nlm.nih.gov/) with the accession number MT900598.

The chloroplast genome of *B. ramiflora* is a typical double-stranded loop structure comprising 161,093 bp in length. It is divided into four regions, including a large single-copy region (LSC) of 89,503 bp, a small single-copy region (SSC) of 18,818 bp, and two inverted repeat regions (IRa and IRb) of 26,386 bp. A total of 128 genes were annotated, including 82 protein-coding genes, 37 tRNA genes, 8 rRNA genes, and 1 pseudogene. The whole chloroplast genome contains 50,480 A bases (31.34%), 51,476 T bases (31.95%), 29,070 G bases (18.05%), and 30,067 C bases (18.66%). The total GC content is 36.71%, with the LSC, SSC, and IR regions of 34.41%, 30.81%, and 42.72%, respectively. Regarding gene function, the 128 genes are divided into four major groups, including genes for self-replication, photosynthesis, unknown function, and other genes.

Twenty other species of Malpighiales were selected to study the phylogenetic relationship between *B. ramiflora* and other Malpighiale species. Among them, seven were Salicaceae, three Phyllanthaceae, five Euphorbiaceae, and two Malpighiaceae. The remaining three species belong to Achariaceae, Erythroxylaceae, or Ctenolophonaceae. *Oxalis corymbose*, a tree species belonging to the Oxalidaceae family (order Oxalidales), was used as an outgroup. The chloroplast genome sequences of 21 species were downloaded from the GenBank (accession numbers are shown in Figure 1). Multiple sequence alignment was performed using MAFFT (Katoh and Standley 2013), whereas RAxML8.2.4 was employed to conduct phylogenetic analysis (Stamatakis 2014). Node support was estimated from the results of 1000 bootstrap replicates. The phylogenetic analysis revealed that *B. ramiflora* is closely related to *Phyllanthus emblica*, *Glochidion chodoense,* and *Phyllanthus amarus*. This study provides valuable genomic data for conservation genetics and future phylogenetic studies of the *B. ramiflora* and Phyllanthaceae family.

**Figure 1.**
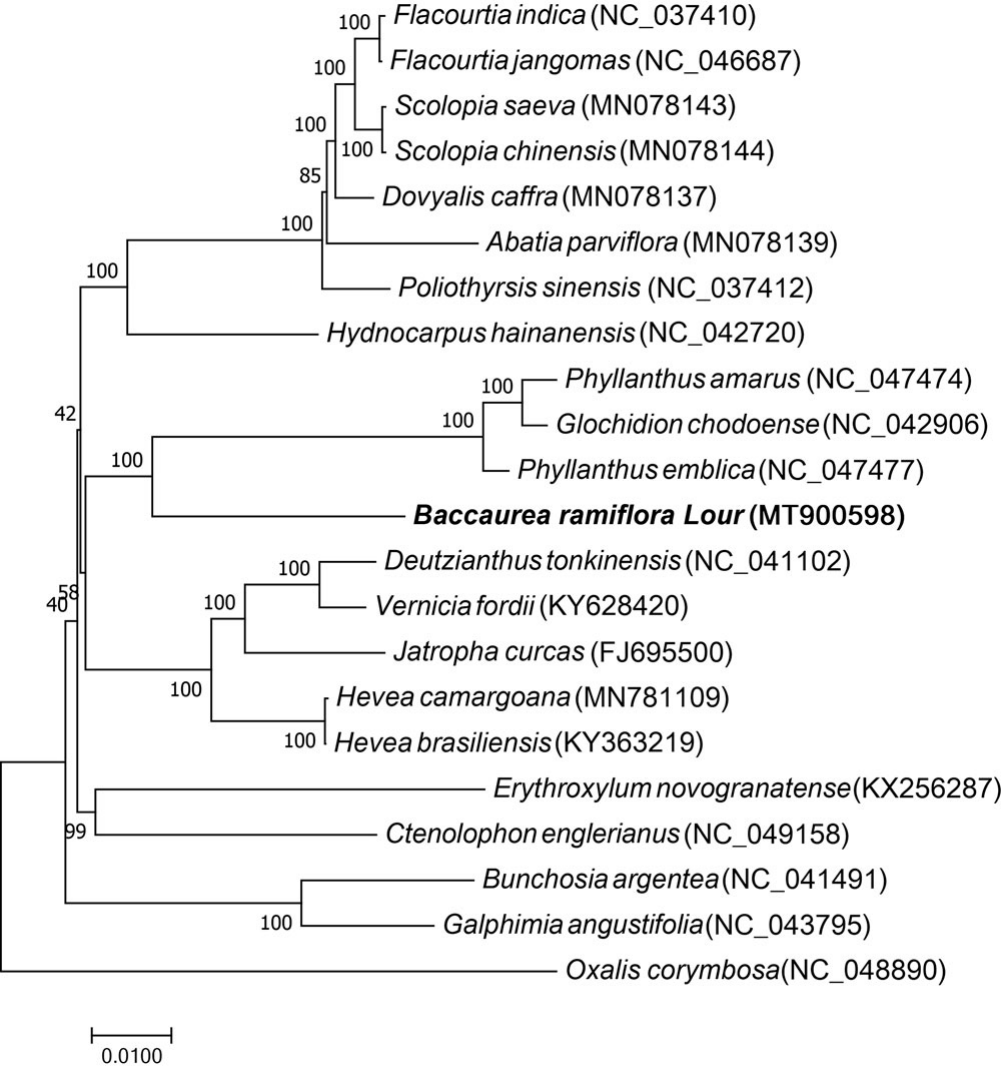
Maximum likelihood tree based on the complete chloroplast genome sequences of *B. ramiflora* and 20 other species of the order Malpighiales. *Oxalis corymbosa* belonging to the order Oxalidales was used as an outgroup.

## Data Availability

The genome sequence data that support the findings of this study are openly available in GenBank of NCBI at (https://www.ncbi.nlm.nih.gov/) under the accession no. MT900598. The associated BioProject, SRA, and Bio-Sample numbers are PRJNA703084, SRR13781754, and SAMN18011299 respectively.

## References

[CIT0001] Goyal AK, Middha SK, Talambedu U. 2020. *Baccaurea ramiflora* Lour.: a comprehensive review from traditional usage to pharmacological evidence. Adv Tradit Med. DOI: 10.1007/s13596-020-00489-9.

[CIT0002] Goyal AK, Mishra T, Sen A. 2013. Antioxidant profiling of Latkan (*Baccaurea ramiflora* Lour.) wine. Indian J Biotechnol. 12(1):137–139.

[CIT0003] Katoh K, Standley DM. 2013. MAFFT multiple sequence alignment software version 7: improvements in performance and usability. Mol Biol Evol. 30(4):772–780. 23329690 10.1093/molbev/mst010 PMC3603318

[CIT0004] Lin YF, Yi Z, Zhao YH. 2003. Chinese Dai medicine colorful illustrations. 1st ed. Kunming, China: Yunnan Nationality Press; p. 158–160.

[CIT0005] Nesa ML, Karim SMS, Api K, Sarker MMR, Islam MM, Kabir A, Sarker MK, Nahar K, Asadujjaman M, Munir MS. 2018. Screening of *Baccaurea ramiflora* (Lour.) extracts for cytotoxic, analgesic, anti-inflammatory, neuropharmacological and antidiarrheal activities. BMC Complem Altern M. 18(1):35. 10.1186/s12906-018-2100-5 PMC5789535 29378554

[CIT0006] Puja SD, Hasan CM, Ahsan M. 2020. *Baccaurea ramiflora*: isolation of aldehydes and *in vitro* biological investigations. Pharm Pharmacol. 11(07):147–157.

[CIT0007] Shi LC, Chen HM, Jiang M, Wang LQ, Wu X, Huang LF, Liu C. 2019. CPGAVAS2, an integrated plastome sequence annotator and analyzer. Nucleic Acids Res. 47(W1):W65–W73. 31066451 10.1093/nar/gkz345 PMC6602467

[CIT0008] Stamatakis A. 2014. RAxML version 8: a tool for phylogenetic analysis and post-analysis of large phylogenies. Bioinformatics. 30(9):1312–1313. 24451623 10.1093/bioinformatics/btu033 PMC3998144

[CIT0009] Talambedu U, Sushil M, Malay B, Prakash L, Arvind G. 2014. Rosmarinic acid, a new polyphenol from *Baccaurea ramiflora* Lour. leaf: a probable compound for its anti-inflammatory activity. Antioxidants. 3(4):830–842. 26785243 10.3390/antiox3040830 PMC4665505

[CIT0010] Wen B, Cai Y. 2014. Seed viability as a function of moisture and temperature in the recalcitrant rainforest species *Baccaurea ramiflora* (Euphorbiaceae). Ann Forest Sci. 71(8):853–861.

